# Reduced Antibiotic Use in Livestock: How Denmark Tackled Resistance

**DOI:** 10.1289/ehp.122-A160

**Published:** 2014-06-01

**Authors:** Sharon Levy

**Affiliations:** Sharon Levy is a freelance science journalist and contributing editor to *OnEarth*, the magazine of the Natural Resources Defense Council.

**Figure:**
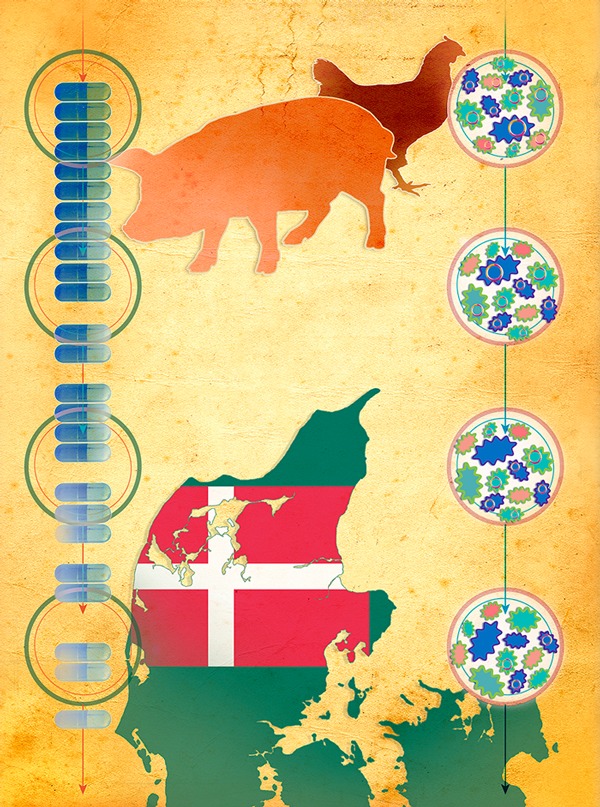
“The bacterial community in the gut of an animal or person is an extremely competitive environment. If you don’t have antimicrobials being used and creating selective pressure for resistance, you’ll get rid of that trait in the long run.” –Yvonne Agersø, Technical University of Denmark © Roy Scott

At the dawn of the antibiotic era, the danger of creating resistant bacteria was already clear. “The time may come when penicillin can be bought by anyone in the shops,” warned Alexander Fleming while accepting his Nobel Prize for the drug’s discovery. “Then there is the danger that the ignorant man may easily underdose himself and by exposing his microbes to nonlethal quantities of the drug make them resistant.”^1^

Toward the end of Fleming’s life, in the 1950s, farmers discovered that feeding low doses of antibiotics to their livestock caused the animals to gain weight faster.^2^ Nobody knows for sure why or how this worked, but the amount of antibiotics used for livestock today is believed to dwarf the amount used in human medicine.^3^

Widespread, indiscriminate use of these drugs is having the impact Fleming predicted. The World Health Organization has named resistance to antimicrobial agents one of the most significant global threats to public health.^4^ In the United States alone, antibiotic-resistant pathogens are conservatively estimated to cause at least 2 million infections and 23,000 deaths each year.^5^

However, one country—Denmark—is leading the way in reversing this trend. Over the past two decades the country has instituted reforms to antibiotic use for livestock that are showing solid progress in reducing the prevalence of resistant bacteria.

## Europe Takes Notice

The introduction of routine antibiotic use in agriculture set the stage for a global mass experiment in the evolution of drug-resistant microbes. “Low-dose, prolonged courses of antibiotics among food animals create ideal selective pressures for the propagation of resistant strains,”^6^ wrote Stuart Levy, a medical doctor and microbiologist at Tufts University, who tracked the phenomenon in a 1974 experiment on a small farm.^7^

Levy’s team found that drug-resistant bacteria quickly came to dominate the intestinal flora of chickens following the introduction of feed laced with oxytetracycline. Within six months, the people living on the farm also carried tetracycline-resistant coliform bacteria, which made up more than 80% of their intestinal microbes. The bacteria carried by both chickens and farmers contained plasmids that conferred traits creating resistance to multiple antibiotics, not only the original drug. The researchers also observed that six months after antibiotics were removed from the chicken feed, most of the workers no longer carried tetracycline-resistant bacteria.

Soon after Levy’s study was published, tetracyclines were banned as growth promoters in Europe.^6^ But in 1994 Frank Aarestrup, a newly graduated veterinarian in Denmark, learned the

prophylactic use of tetracyclines was on the rise in his country—accompanied by an increase in resistant bacteria carried by livestock. Aarestrup was worried by the trend, and even more alarmed when he realized it was being driven by a profit motive among Danish veterinarians, many of whom earned as much as a third of their income by selling antibiotics to farmers.

When he investigated further, he found that the amount of other antibiotics still being prescribed for growth promotion in pigs and poultry far outweighed therapeutic use of the drugs. About 90% of antibiotics given to poultry were administered at low doses for growth promotion.^8^

Aarestrup, who is now a professor at the Technical University of Denmark (DTU), and his colleagues undertook their own studies of antibiotic-resistant bacteria in the feces of healthy chickens and pigs. Their work uncovered a clear relationship between the use of the antibiotic avoparcin and the widespread occurrence of resistant bacteria.^8^^,^^9^ Avoparcin is a glycopeptide with a chemical structure similar to vancomycin, a treatment of last resort in human patients with life-threatening infections.^10^ In 1995 Aarestrup reported that 80% of chickens sampled on conventional farms (where avoparcin was used as a growth promoter) carried bacteria resistant to vancomycin. None of the chickens sampled on organic farms, where no growth promoters were used, carried vancomycin-resistant microbes.^11^

As early as the 1960s, European countries had banned the use of any antibiotic important in human medicine as a growth promoter.^12^ However, the ban covered only specific drugs, such as vancomycin, not chemical analogs like avoparcin. Avoparcin was approved as a growth promoter in Europe in the 1970s and was widely used in livestock.^13^ In the United States, meanwhile, avoparcin was never approved for any use in agriculture, but vancomycin was being commonly administered in hospitals, contributing to the rise of vancomycin-resistant enterococci (VRE). In hospital patients already weakened by other health problems, VRE can cause serious infections. In the United States, 20,000 hospital patients contract VRE infections each year, and 1,300 of them die.^5^

By the 1990s, VRE was much more common among the European general population compared with the U.S. population. It appears that VRE strains from livestock entered the general community in Europe whereas these strains remained restricted to hospitals in the United States.^12^ A 1997 study of long-time vegetarians versus meat-eaters in the Netherlands revealed that none of the vegetarians carried VRE, while 20% of the meat-eaters did.^14^ That year, the European Union banned all uses of avoparcin.^15^

## Success for Denmark

In Denmark, the drive to preserve antibiotics for human use revolutionized livestock management during the 1990s and 2000s. The country drastically limited how much veterinarians could profit from the sale of antibiotics in 1995, and in the same year became the first European country to ban all uses of avoparcin. By 1999, all nontherapeutic use of antibiotics in pigs was outlawed—a huge change in a nation that is the world’s leading exporter of pork.^12^

In most cases, halting the nontherapeutic use of antibiotics in livestock leads to a significant decrease in resistant microbes in animals and meat within a year or two—as Levy’s work suggested decades ago. In other cases, depending on the drug involved and other factors, resistance can fade more slowly. “We’ve looked at this in poultry and in pig production,” says Yvonne Agersø, a senior researcher at DTU. “The bacterial community in the gut of an animal or person is an extremely competitive environment. If you don’t have antimicrobials being used and creating selective pressure for resistance, you’ll get rid of that trait in the long run.”

Data from Denmark show a marked decline in levels of VRE in pigs since the 1995 avoparcin ban.^16^ A study in the Netherlands found that within two years of banning avoparcin, the prevalence and numbers of VRE decreased significantly in the fecal flora of both food animals and healthy humans.^17^ And a significant decline in resistant bacteria was documented two years after Danish pig farmers voluntarily stopped using cephalosporins in 2009.^18^

One of the most striking aspects of Denmark’s transformation in antibiotics policy is that it reportedly has had little negative impact on the nation’s pork industry.^8^^,^^12^ From 1992 to 2008, antibiotic use per kilogram of pig raised in Denmark dropped by more than 50%. Yet overall productivity increased. Production of weaning pigs increased from 18.4 million in 1992 to 27.1 million in 2008.^19^ Pig mortality began increasing in 1994 but fell sharply after 2004 and by 2008 was similar to 1992 levels.^19^

According to Niels Kjeldsen, a veterinarian with the Danish Agriculture and Food Council, the cost of raising pigs has gone up by about ¤1 per animal, from birth to slaughter, since the ban.

**Figure:**
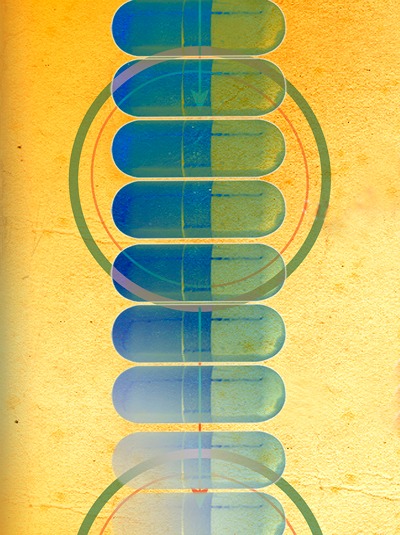
Antibiotics in U.S. Agriculture 2011 U.S. livestock producers purchased 29.9 million pounds of antimicrobials,^37^ including millions of pounds of drugs that are prohibited for nontherapeutic agricultural use in the European Union. It is unknown how much of this was used for growth promotion and disease prevention, although in 2001 the Union of Concerned Scientists estimated that nontherapeutic uses accounted for 93% of the antibiotics used in U.S. livestock.^38^ The United States uses more antibiotics per kilogram of meat and poultry produced than any other developed country.^39^

“We have more efficient production and less disease,” says Jørgen Schlundt, director of the National Food Institute at DTU. Many Danish farmers now allow piglets to stay with their mothers for a longer period, which allows them to build their immune systems naturally. Piglets separated from their mothers very early in life are much more susceptible to infection.^20^

Schlundt emphasizes that close monitoring of antibiotic sales and use is an essential part of the Danish system. “We started monitoring even before we introduced the restrictions on antibiotic use, so we would have baseline data,” he says. “We track the amount of antibiotics used in animals and in humans, and monitor resistance in pathogens and indicator organisms.” This information was needed both to enable the government to intervene with the few farmers who continued to overuse antibiotics, and to convince the agricultural community that the ban was effective as a public health strategy. The evidence from Denmark, and elsewhere in Europe, has been so convincing that the entire European Union banned the use of growth promoters in 2006.^21^

The Danish system has succeeded through collaboration between the agriculture industry, veterinarians, human health researchers, and the government. The shift was made easier by Denmark’s farming culture, where farmers’ organizations developed from a system in which dairies and slaughterhouses were owned by farmer co-ops. Schlundt says this seems to give farmers a greater sense of responsibility for the impacts of food production practices.

“When I talk to people from the U.S. about this,” he notes wryly, “I have to start by saying that Danes are not communists.” The antibiotic restrictions in Denmark are a science-based policy change, he says, well-supported by data from multiple sources. Schlundt points out that this kind of thinking is hardly foreign to the United States. “The Danish researchers doing epi work on these issues in Denmark all had some level of training at [the U.S. Centers for Disease Control and Prevention],” he explains. “All Danish work on risk assessment is directly impacted by U.S. thinking.”

## The U.S. Situation

Meanwhile, U.S. policy on antibiotic use in livestock has remained in a kind of limbo for more than 35 years.^22^ In 1977 the U.S. Food and Drug Administration (FDA) proposed banning tetracyclines and penicillins as additives in livestock feed.^23^ A congressional committee asked the agency for additional data, which it provided. But no further action was taken.

In 1999 and again in 2005, environmental and health groups petitioned the FDA to move forward with its 1977 proposal and to extend the prohibition to other kinds of antibiotics.^24^ After a lawsuit was filed in 2011, the agency denied the citizen petitions on the grounds that formal withdrawal proceedings for controversial substances would take inordinate amounts of time and resources, and that the animal pharmaceutical industry had indicated it was “generally responsive” to the prospect of voluntary changes in antibiotic use.^25^^,^^26^ A coalition of public interest groups led by the Natural Resources Defense Council (NRDC), which filed the lawsuit, challenged the agency’s denial of the petitions, alleging the decision was not based on science and safety considerations.^27^

In March 2012 federal judge Theodore Katz directed the FDA to move forward on the 1977 proposal to ban the use of penicillins and tetracyclines in animal feed for growth promotion unless drug manufacturers could prove that such use is safe.^28^ Three months later the judge ruled in favor of the plaintiffs and ordered the FDA to move forward with the ban. “If … the drug industry intends to comply with the voluntary program, then it is unclear why the industry would contest formal withdrawal notices or require time consuming hearings,” wrote Judge Katz.^29^

The FDA appealed Katz’s decision,^30^ and the case continues to work its way through the legal system. In December 2013 the agency finalized its voluntary guidelines, which ask drug companies to remove growth-promotion claims from their labels and prevent antibiotics administered in food and water from being sold over the counter for prophylactic use (such use would require a veterinarian’s prescription).^31^ “FDA is currently working in collaboration with other agencies, including the [U.S. Department of Agriculture and the Centers for Disease Control and Prevention], to explore approaches for enhancing current data collection efforts in order to measure the effectiveness” of the voluntary guidelines, says Siobhan DeLancey of the FDA Office of Media Affairs.

For many public health advocates, this measure doesn’t go nearly far enough. “The voluntary approach is not likely to work,” says Avinash Kar, a staff attorney for the NRDC. “There’s a huge loophole: The FDA’s guidance endorses the use of antibiotics for disease prevention,” although it urges that such use be “judicious.”^31^

That same loophole remains a problem in much of Europe, years after the EU ban on antibiotics as growth promoters. Schlundt says mass administration of low-dose antibiotics continues for the stated purpose of disease prevention, despite a lack of solid evidence that dosing whole herds this way is a reliable prophylactic. (Low-dose antibiotics can sometimes improve the feed efficiency in nursery pigs—that is, the amount of food consumed by animals per kilogram of weight gained—and increase productivity in chickens somewhat, but often not enough to offset the expense of the drugs.^32^)

When overall antibiotic use did not decrease in the Netherlands following the ban, the Dutch government began to impose fines for overuse of antibiotics in 2009; veterinary consumption of the drugs subsequently dropped by more than 50% in the course of three years.^33^ In the Netherlands, as in Denmark, change was made possible by close tracking of drug sales and use, Schlundt says.

## Surveillance Is Key

The United States lacks anything close to the extensive monitoring system in place in Denmark. “We’re concerned with the lack of surveillance,” says Gail Hansen, an expert on human health and industrial farming at the Pew Charitable Trusts.

The only publicly available data collected in the United States give sales figures for total amounts of antibiotics used on food animals nationwide; the kind of information Danish researchers cite as essential to their system—who is administering what amounts of antibiotic to what animals—is unavailable. The new voluntary guidelines from the FDA also don’t mention monitoring. “We’ve asked the agency repeatedly how they plan to monitor this and not gotten meaningful answers,” says Hansen.

A small group of senators and Congress members have been trying to address the issue through legislation. Representative Louise Slaughter (D–NY) and Senator Dianne Feinstein (D–CA) have long supported bills they call the Preservation of Antibiotics for Medical Treatment Act (H.R. 1150)^34^ and the Preventing Antibiotic Resistance Act (S. 1256),^35^ respectively. The bills have failed to make their way out of committee onto the floor of either house, although both continue to gain new sponsors. In October 2012 Representative Henry Waxman (D–CA) introduced the Delivering Antimicrobial Transparency in Animals Act (H.R. 820),^36^ which would require large-scale producers of poultry, swine, and other livestock to submit detailed annual reports to the FDA on the type and amount of antibiotics contained in the feed given to their animals. That bill also made no progress.

“The industrial farm system in the U.S. has grown up with antibiotics,” notes Hansen. “But throwing antibiotics at a problem and calling it prevention almost never works.”

Chronic, low-dose administration, she says, is the worst possible way to use the drugs that transformed medicine in the twentieth century. Unless we can change our ways, the twenty-first century may witness the end of that medical miracle. Denmark’s experience shows a practical way of moving toward a different future—one that holds both a healthy livestock industry and viable antibiotic therapies for people who need them.

**Figure:**
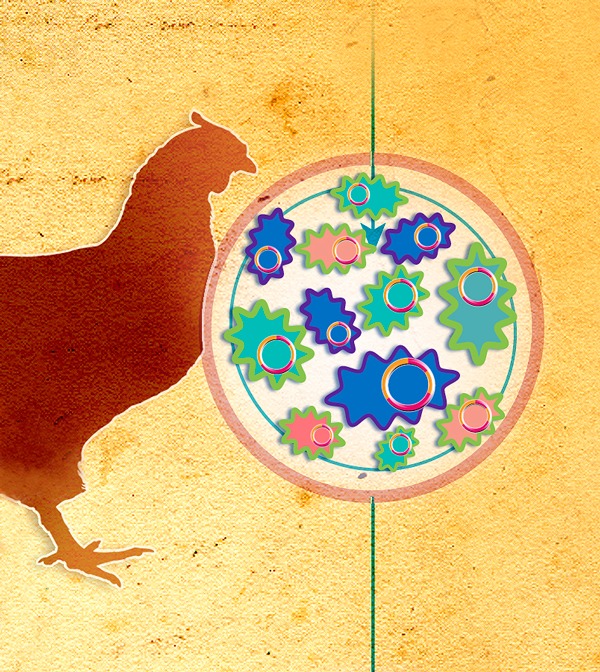
The Need for International Standards Unfortunately, Denmark’s comprehensive reform of antibiotic use in agriculture doesn’t necessarily mean Danes are safe from antibiotic-resistant pathogens carried in animals or meat. That’s illustrated by recent work^40^ by Yvonne Agersø tracking the emergence of bacteria that carry a gene for the production of extended spectrum β-lactamase (ESBL) enzymes, which confer resistance to both penicillins and cephalosporins. Cephalosporins have been widely used as growth promoters in chickens in some parts of the world, but were never used on Danish poultry. Yet recent data show a dramatic rise in the incidence of ESBL-producing Escherichia coli bacteria in chicken meat sold in Denmark. In 2012 testing showed that 61% of samples of imported chicken were contaminated with ESBL-producing E. coli, but the same kinds of microbes also were identified in 36% of samples from poultry raised in Denmark, even though the chickens had never received cephalosporins.^41^ Agersø and her colleagues tracked the resistant microbes back through generations of birds.^40^ The grandparents of the contaminated Danish chickens had been imported from Scotland, where they were treated with cephalosporins very early in life, and resistant bacteria passed from one generation to the next. A Swedish team recently reported similar findings for chickens in that country.^42^ The findings point up the need for international standards restricting the agricultural use of antibiotics.
